# Molecular characterization, phylogenetic and *in silico* sequence analysis data of trehalose biosynthesis genes; *otsA* and *otsB* from the deep sea halophilic actinobacteria, *Streptomyces qinglanensis* NIOT-DSA03

**DOI:** 10.1016/j.dib.2021.106727

**Published:** 2021-01-21

**Authors:** Balakrishnan Meena, Lawrance Anburajan, Nambali Valsalan Vinithkumar, Ramalingam Kirubagaran, Gopal Dharani

**Affiliations:** a Atal Centre for Ocean Science and Technology for Islands, National Institute of Ocean Technology, Ministry of Earth Sciences, Government of India, Port Blair-744103, Andaman and Nicobar Islands, India; b Marine Biotechnology Division, Ocean Science and Technology for Islands Group, National Institute of Ocean Technology, Ministry of Earth Sciences, Government of India, Chennai 600100, Tamil Nadu, India

**Keywords:** Deep sea, Halophilic actinobacteria, Trehalose genes, Compatible solutes, Osmolytes

## Abstract

Trehalose, a non-reducing disaccharide (α-D-glucopyranosyl-(1→1)-α-D-glucopyranoside) is a natural compound, which serves as a protective substance in halophilic bacterial cells. Trehalose biosynthesis genes (*otsA* and *otsB*) were PCR amplified from the genomic DNA of deep sea actinobacteria, *Streptomyces qinglanensis* NIOT-DSA03. The amplified genes were cloned and nucleotide sequences were determined. *In silico* sequence and phylogenetic analysis of nucleotides and amino acids of *otsA* and *otsB* sequences of *S. qinglanensis* were also determined. The experimental data described in this study will be helpful to develop a recombinant expression system to produce trehalose for biotechnological applications.

**Specifications Table**

**Table:** 

Subject	Applied Microbiology and Biotechnology
Specific subject area	Biotechnology and Bioinformatics
Type of data	Images, Figures
How data were acquired	Molecular cloning, BLAST program of NCBI (http://www.ncbi.nlm.nih.gov), CLUSTALW program, GeneDoc program (https://genedoc.software.informer.com/2.7/), BioEdit 7.05 program (www.mbio.ncsu.edu/BioEdit/), PROTEAN program, MODELLER program
Data format	Raw and Analysed data
Parameters for data collection	Data were collected using polymerase chain reaction studies, nucleic acid electrophoresis through agarose gels, molecular cloning, DNA sequencing and *in silico* analysis.
Description of data collection	Sequencing results revealed that *otsA* and *otsB* genes contains 1278 bp and 879 bp long ORF encoding 425 and 292 amino acids, respectively. *In silico* sequence and phylogenetic analysis of nucleotides and amino acids revealed that the *otsA* and *otsB* sequences of *Streptomyces qinglanensis* NIOT-DSA03 were conserved in many eubacteria.
Data source location	National Institute of Ocean Technology Port Blair India (12 °12.90’N, 093 °48.92’E)
Data accessibility	Data is available with this publication

## Value of the Data

• The dataset describes the importance of major osmolyte, trehalose in protecting the proteins and cellular membranes in prokaryotes from inactivation or denaturation by the environmental stress.
• The dataset provides information about the osmolyte in *Streptomyces qinglanensis* NIOT-DSA03 and its application in derma-pharmacy industries.
• In this data we have provided the detailed information regarding the gene sequence and its protein structure. This data may be used for further heterologous gene expression studies.

## 1 Data Description

**Fig. 1:**
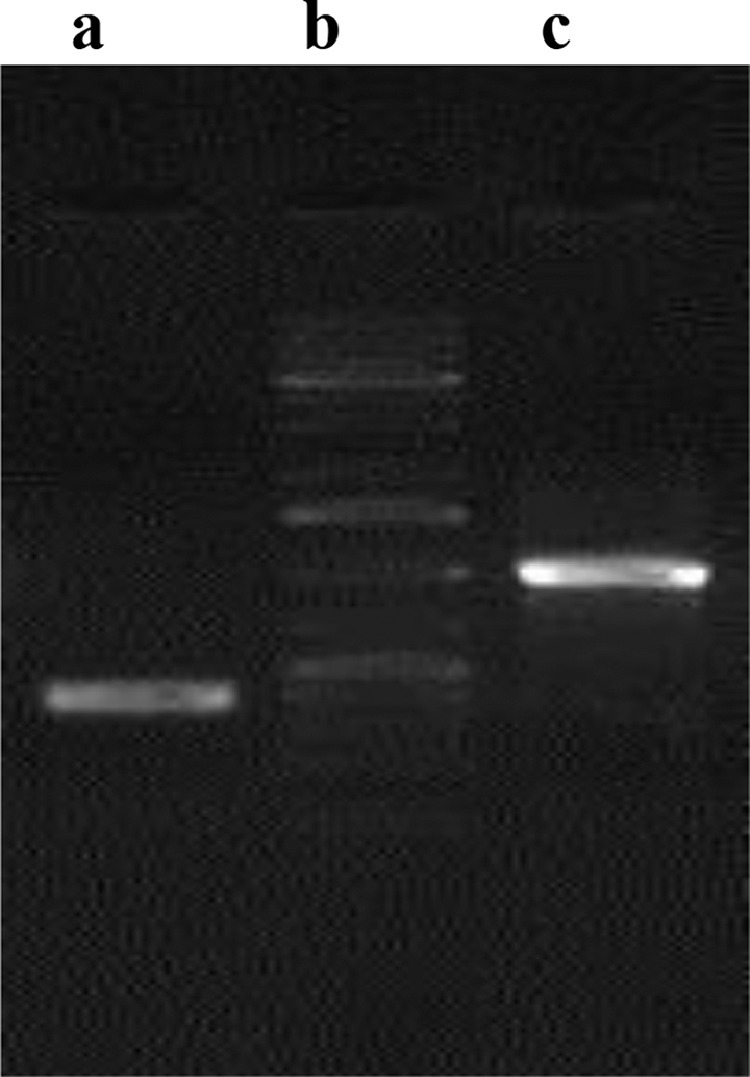
Agarose gel electrophoresis of *otsA* and *otsB* gene amplicons. Lane a: *otsB* amplicon 879 bp, Lane c: *otsA* amplicon 1,278 bp, Lane b: 1 kb DNA ladder.

**Fig. 2:**
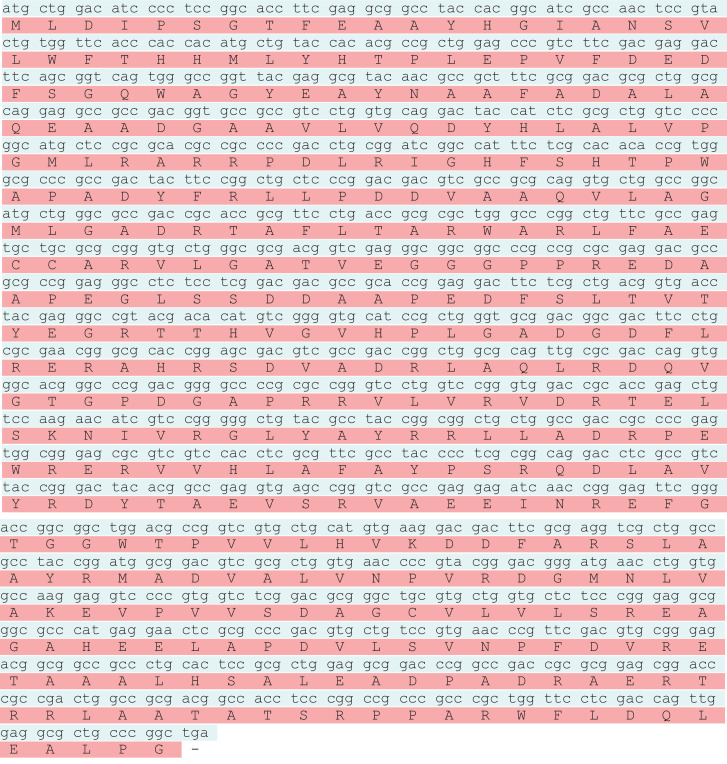

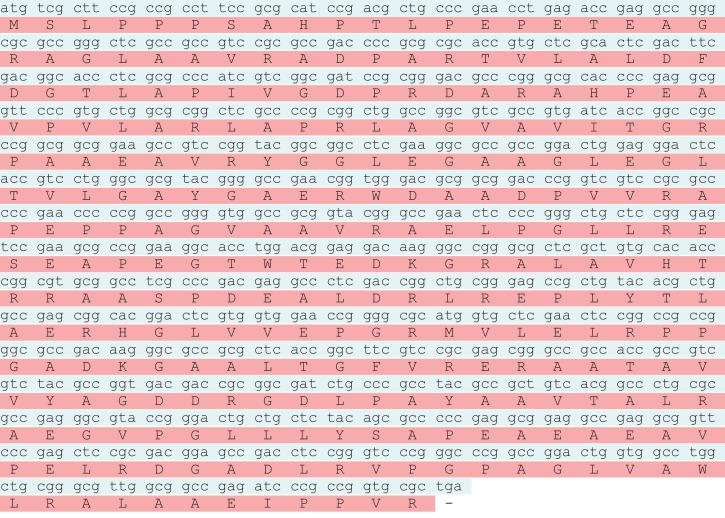
(a) Nucleotide sequences of *otsA* gene in *Streptomyces qinglanensis* NIOT-DSB03. (b) Nucleotide sequences of *otsB* gene in*Streptomyces qinglanensis* NIOT-DSB03.

The *otsA* and *otsB* genes encode the, α-trehalose-phosphate synthase and trehalose-6-phosphate phosphohydrolase respectively. Together these proteins constitute the trehalose biosynthetic pathway. The trehalose biosynthesis genes *otsA* and *otsB* were PCR amplified and are encoded by polynucleotides of 1278 bp and 879 bp (Fig. 1). The *otsA* and *otsB* genes encodes proteins of 425 and 292 amino acids with calculated molecular masses of 46625, 30228 Daltons (Fig. 2a & b). After PCR amplification, the products were purified from the agarose gel and cloned into pDrive cloning vector. The white colonies were selected and screened for the presence of insert by PCR amplification using specific primers, which gave specific product. The recombinant transformants of *otsA* and *otsB* genes were also confirmed by double digestion with *Sac* I and *Bam* HI restriction enzymes, which released full gene along with flanking region of the vector. The *otsA* and *otsB* sequences generated in this study have been deposited in the GenBank database with the accession numbers MN017301 and MN023141.

The *otsA* and *otsB* sequences from *S. qinglanensis* NIOT-DSA03 were analyzed with reported amino acid sequences of other actinobacteria viz. *S.venezuelae,* (GenBank accession no. LN881739.1), *S. lIncolnensis,*(CP016438), *S. clavuligerus,* (CP016559), *S. fradiae,* (CP032266), *Streptomyces* sp.*,* (CP029188), *S. alfalfa,* (CP015588), *S. venezuelae,* (LN881739), *S. clavuligerus*, (CP016559), using Clustal W program [1].

**Fig. 3:**
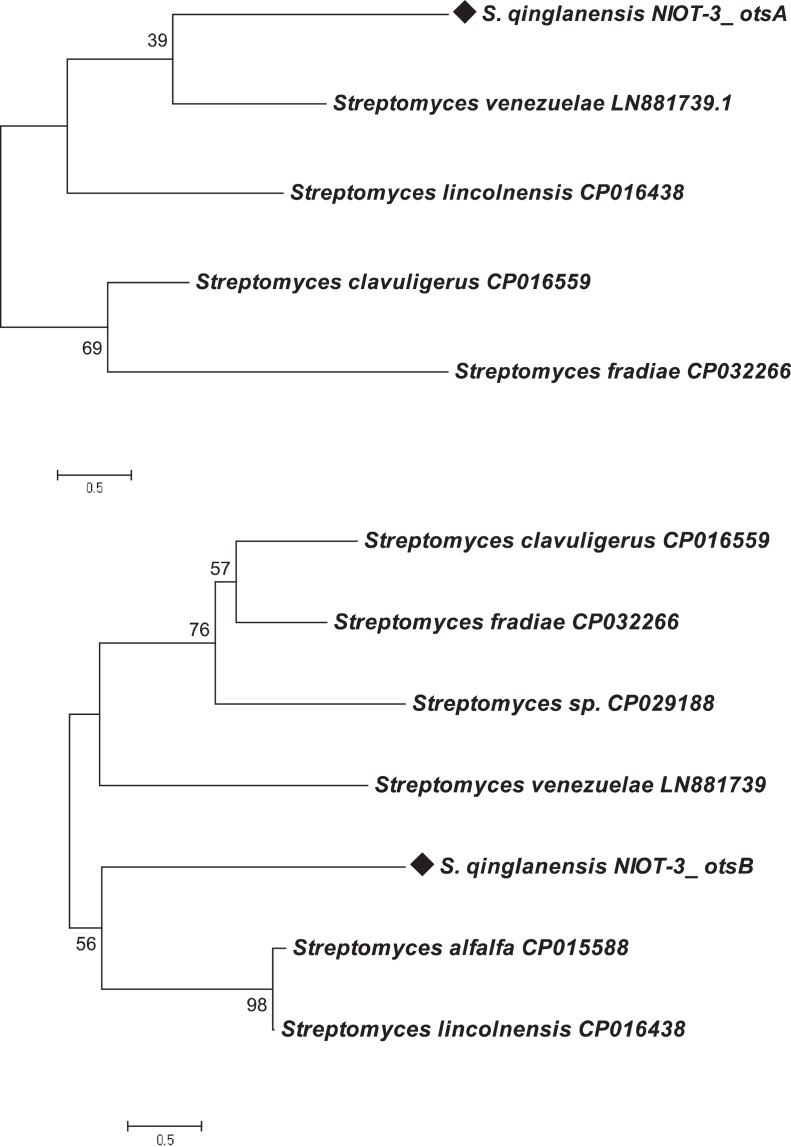
(a) Phylogenetic tree analysis of *otsA* amino acid using MEGA program. (b) Phylogenetic tree analysis of *otsB* amino acid using MEGA program.

The phylogenetic tree at nucleotide and amino acid level of *otsA* revealed the phylogenetic similarity of *otsA* gene from *S. qinglanensis* NIOT-DSA03 with other organisms. The bacterial species switched to different clusters for *otsA* gene at nucleotide and amino acid level indicating divergence among the organisms and the degree of divergence in the sequences. *S. qinglanensis, S. venezuelae* and *S. lIncolnensis* were grouped in the same cluster in both the phylogenetic trees (Fig. 3a). The phylogenetic tree of nucleotide and amino acid sequences of *otsB* gene also revealed the grouping of *S. qinglanensis, S. alfalfa* and *S. lIncolnensis* in a single cluster as that of *otsB*. In phylogenetic tree analysis, a diverged mode of clustering was observed (Fig. 3b).

On phylogenetic analysis, the *otsA* and *otsB* genes of *S. qinglanensis* was found to be highly conserved among the bacterial species. The *otsB* gene was found to have highest similarity between bacterial species compared to the *otsA* gene. Based on phylogenetic analysis, *S. qinglanensis* and *S. lincolnensis* were found to be clustered together for *otsA* and *otsB* genes. The genes involved in the biosynthesis of trehalose in *S. qinglanensis* and *S. venezuelae* are comparatively well conserved compared to other bacteria both at nucleotide and amino acid level [2].

**Fig. 4:**
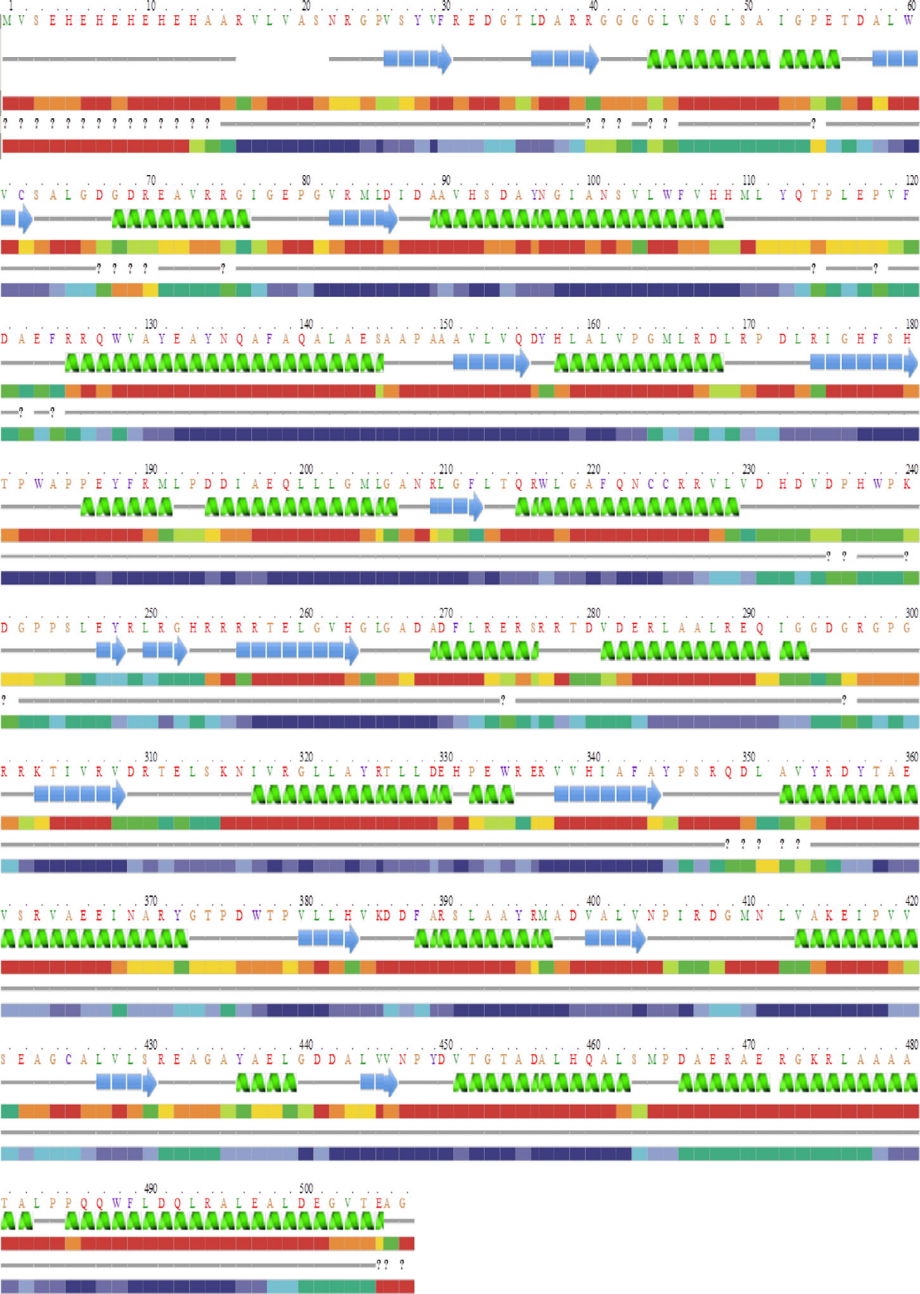

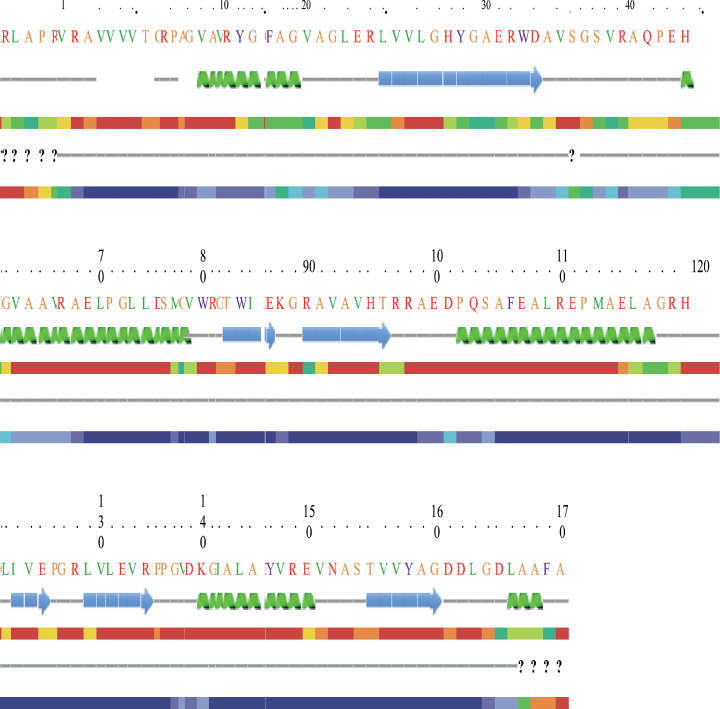
(a) Secondary structure prediction of *otsA* gene (Chou-Fasman and Garnier-Robson algorithms for predicting alpha, beta and turn regions) using PROTEAN program. (b) Secondary structure prediction of *otsB* gene (Chou-Fasman and Garnier-Robson algorithms for predicting alpha, beta and turn regions) using PROTEAN program.

Prediction of secondary structure was performed with the PROTEAN program (Discovery studio 3.5). The secondary structure of *otsA* and *otsB* proteins were predicted to have the alpha-helical structure with maximum hydrophilic molecules. The prediction analysis also revealed the presence of many acidic amino acids; regions with high antigenicity and very high backbone chain flexibility. Upon analysis of *otsA* protein, the predicted charge at pH 7.0 was "+9.54" with the isoelectric point of 5.82. Common amino acids include 14% glutamic acid, 10% leucine, 11% each of alanine, threonine, valine and 17% each of phenylalanine, glycine, proline and glutamine (Fig. 4a). In *otsB* protein, the predicted charge at pH 7.0 was "+20.14" with the isoelectric point of 5.64. The amino acid composition includes 52% glycine, 41% glutamic acid, 37% leucine, 39% threonine and 29% lysine (Fig 4b). This prediction result also showed considerable similarity with the reported trehalose biosynthesis enzymes from actinobacteria.

**Fig. 5:**
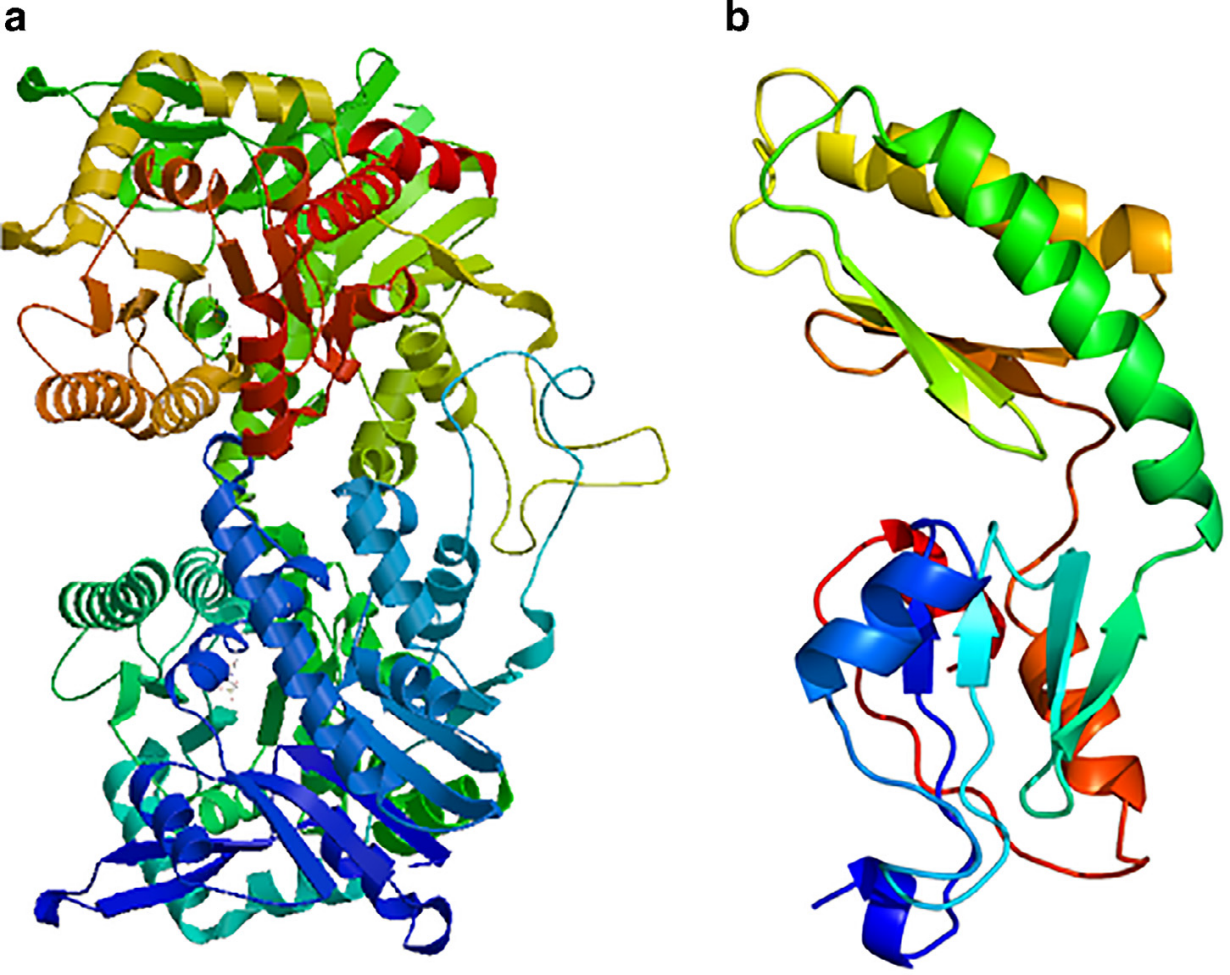
(a) Three dimensional structure prediction of *otsA* gene using MODELLER program. (b) Three dimensional structure prediction of *otsB* gene using MODELLER program.

Three dimensional structure prediction of the trehalose synthase enzyme suggests that the tertiary structure was highly compatible with the secondary structure prediction analysis. The structure was validated using Ramachandran plot and the plot suggested that none of the residues were present in the disallowed region. This deduces that the modelled structure shares high level of similarity with the structures that have been already reported. Homology analysis of trehalose synthase enzyme with Protein Data Base (PDB) revealed the maximum of 100% and minimum of 21% identity with the PDB templates (Fig. 5a & b).

## 2 Experimental Design, Materials and Methods

### 2.1 Bacterial strain, growth conditions, DNA isolation and plasmids

*S. qinglanensis* NIOT-DSA03 was isolated from the deep sea sediment sample obtained during the cruise of the Barren Island, Andaman and Nicobar (A & N) Islands in the ocean research vessel Sagar Manjusha. Using box cores at a depth of 1,840 m (12 ° 12.90 'N, 093 ° 48.92′ E), sediment samples were collected from the seafloor. In the ISP 1 medium, the isolate was grown aerobically and the genomic DNA was isolated following the modified Kutchma et al., procedure. [3]. Using the universal Eubacterial primers, 16S F (5′-ACTCAAGGAATTGACGG-3′) and 16S R (5′-TACGGCTACCTGTTACGACTT-3′), the 16S rDNA was amplified by polymerase chain reaction. According to the instructions of the manufacturer in the InsTAclone PCR Cloning Kit, the 16S rDNA amplicon was cloned into a T/A cloning vector (MBI Fermentas, USA). Using the dye termination process, DNA sequencing was carried out on an ABI PRISM 377 genetic analyzer (Applied Biosystems, USA). In a homology search with the available sequences in GenBank using BLAST provided for pair identities by NCBI, the acquired 16S rDNA sequences were used. As the host strain for transformation in cloning, the *Escherichia coli* JM109 strain was used and the plasmid pDrive was used as the cloning vector.

### 2.2 PCR amplification of trehalose biosynthesis genes

Trehalose biosynthesis genes, *otsA* and *otsB* were individually amplified by PCR using gene-specific primers designed by a program available at http://frodo.wi.mit.edu/primer3 otsA F (5′-ATGCTGGACATCCCCTCC-3′) and otsA R (5′-TCAGCCGGCAGCCCTC-3′) and otsB F (5′-ATGTCGCTCCGCCCGCCCC-3′) and otsB R (5′-CAGCGCCGGGATC-3′). The final volume of PCR was 50 μl, each containing 0.5 μM of forward and reverse primers; 1.0 μl of crude genomic DNA; 200 μM of dNTP; 1 × *Pfu* buffer; 2.5 mM MgSO4; 1.0 U of *Pfu* DNA polymerase (MBI Fermentas, USA) and remaining autoclaved Milli Q water. In the Master Cycler (Eppendorf, Germany), amplification was carried out under the following conditions; initial denaturation at 94 °C for 3 min, followed by 30 repeated cycles at 94 °C for 30 sec, 50 °C for 1 min, 72 °C for 2 min and final extension at 72 °C for 5 min. The PCR amplicons were analyzed on 1.5 percent agarose gel along with 100 bp DNA ladder (MBI Fermentas) and recorded in gel documentation system (UVP BioSpectrum Imaging system, USA).

### 2.3 Molecular cloning of trehalose genes

The *otsA* and *otsB* gene amplicons were purified by the MinElute Gel purification kit (Qiagen, Germany) and as directed by the manufacturer, cloned into pDrive (Qiagen). The gene constructs of pDrive-*otsA* and *otsB* have been transformed into *E. coli* JM109 (recA1, endA1, gyrA96, thi-1, hsdR17 (rk-mk+), e14-(mcrA-), supE44, relA1, Δ (lac-proAB)/F '[traD36, proAB+, lacIq, lacZ Δ M15]). White colonies were selected for PCR amplification with M13f-M13r (MBI Fermentas) vector primers, and clones with the correct insert were selected for alkali lysis process plasmid isolation as measured by size and correct orientation [4].

### 2.4 Characterization of recombinant plasmids

The recombinant plasmids with pDrive-*otsA* and *otsB* gene constructs were double digested with *Sac*I and *Bam* HI enzymes. The restriction digestion was carried out with a final volume of 20 μl comprising 2 μl of each recombinant plasmid; 1 × enzyme buffer; 5 U/μl of each restriction enzyme and the remaining Milli Q autoclaved water. In a water bath, reaction mixtures were incubated overnight at 37  °C and the digested products were analyzed on 1.5 percent agarose gel along with 100 bp DNA ladder and documented in the gel documentation system (UVP BioSpectrum Imaging system, USA). The restriction digested trehalose biosynthesis genes were gel eluted, purified and sequenced on an ABI PRISM 377 genetic analyzer (Applied Biosystems Inc., USA).

### 2.5 In silico sequence analysis of trehalose biosynthesis genes

The nucleotide sequences acquired were compared with database sequences using NCBI's BLAST (http://www.ncbi.nlm.nih.gov) program and were aligned and clustered using CLUSTALW [5]. In order to measure the percentage identities between nucleotide and amino acid sequences, alignments were imported into the GeneDoc program (https://genedoc.software.informer.com/2.7/) and the BioEdit 7.05 program (www.mbio.ncsu.edu/BioEdit/). Using the ProtParam tool (http://www.expasy.org/tools/protparam.html), the molecular masses and theoretical pI values of the polypeptides were predicted. Prediction of secondary structure was performed with the PROTEAN program (DNASTAR, Inc., Madison). The three dimensional structure was predicted through homology modelling approach using MODELLER program (Discovery Studio Modeling Environment 4.0, San Diego: Accelrys Software Inc., 2013).

## Declaration of Competing Interest

The authors declare no competing interests.

## References

[1] Kumar S., Tamura K., Nei M. (2004). MEGA3: integrated software for molecular evolutionary genetics analysis and sequence alignment. Brief. Bioinform..

[2] Schiraldi C., Di Lernia I., De Rosa M. (2002). Trehalose production: exploiting novel approaches. Trends Biotechnol..

[3] Kutchma A.J., Roberts M.A., D.B.Knaebel D.L.Crawford (1998). Small-scale isolation of genomic DNA from Streptomyces mycelia or spores. Biotechniques.

[4] Sambrook J, Russell D.W. (2001). A Molecular Cloning: A Laboratory Manual.

[5] Thompson J.D, Gibson T.J, Plewniak F, Jeanmougin F, Higgins D.G. (1997). The CLUSTAL X windows interface: flexible strategies for multiple sequence alignment aided by quality analysis tools. NucleicAcids Res..

